# Supplementary material for: Air Pollution Detection Using a Novel Snap-Shot Hyperspectral Imaging Technique
Source: Sensors (Basel). 2022 Aug 19;22(16):6231. doi: 10.3390/s22166231 (PMC9416790; doi:10.3390/s22166231)
Supplement: Supplementary file 1 [file sensors-22-06231-s001.zip › sensors-1861006-supplementary.pdf]

Supplementary Material

Air Pollution Detection Using a Novel Snap-Shot Hyperspectral Imaging Technique

Arvind Mukundan [1], Chia-Cheng Huang [1], Ting-Chun Men [1], Fen-Chi Lin [2,*] and Hsiang-Chen Wang [1,*]

[1] Department of Mechanical Engineering, Advanced Institute of Manufacturing with High Tech Innovations (AIM-HI) and Center for Innovative Research on Aging Society (CIRAS), National Chung Cheng University, 168, University Rd., Min Hsiung, Chiayi City 62102, Taiwan; d09420003@ccu.edu.tw (A.M.); jerry85418@gmail.com (C.-C.H.); asd19970810@gmail.com (T.-C.M.)

[2] Ophthalmology, Kaohsiung Armed Forces General Hospital, 2, Zhongzheng 1st. Rd., Lingya District, Kaohsiung City 80284, Taiwan

* Correspondence: eses.taiwan@gmail.com (F.-C.L.); hcwang@ccu.edu.tw (H.-C.W.)

S1. Training Results

Table S1. Classification Training Results of all the three modes.

Final accuracy(validation data):			
(a)RGB	precision	recall	fl - sore
pm2.5_good	0.8123	0.9719	0.885
pm2.5_moderate	0.8417	0.5805	0.6871
pm2.5_severe	0.8841	0.8756	0.8798
(b)PCA	precision	recall	fl-sore
pm2.5_good	0.9336	0.8386	0.8835
pm2.5_moderate	0.7606	0.8218	0.7901
pm2.5_severe	0.8527	0.9139	0.8822
(c)3D-CAE	precision	recall	fl-sore
pm2.5_good	0.4273	0.8339	0.5651
pm2.5_moderate	0.2593	0.0380	0.0664
pm2.5_severe	0.2987	0.1179	0.1691

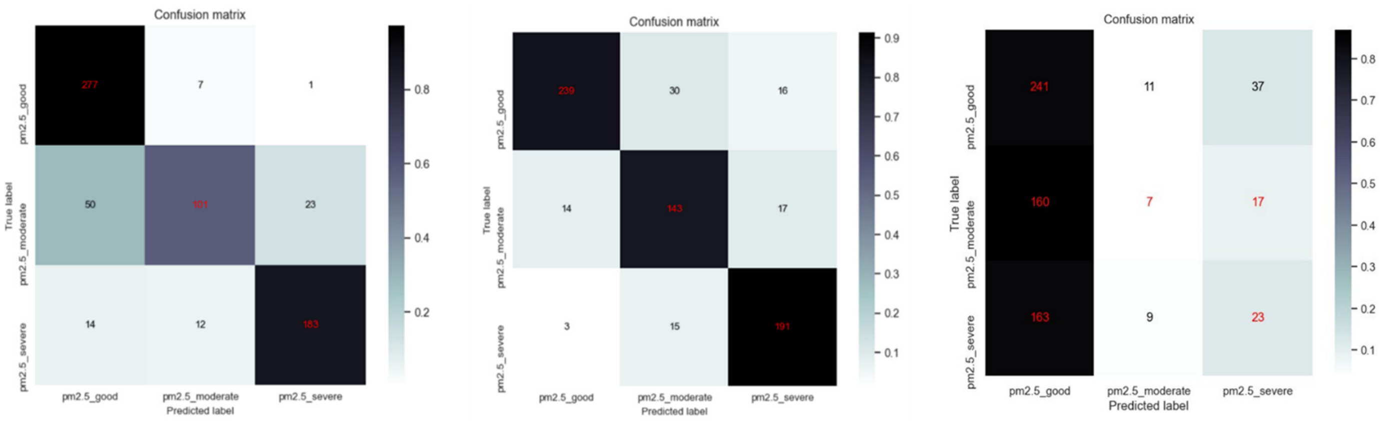

Figure S1. Confusion matrix after training all three models.

Table S2. Definitions of the terms in Confusion Matrix.

Metrics	Abbreviations	Definitions
True Positive	TP	A test result that correctly indicates the presence of a condition or characteristic
True Negative	TN	A test result that correctly indicates the absence of a condition or characteristic
False Positive	FP	A test result that wrongly indicates that a particular condition or attribute is present
False Negative	FN	A test result that wrongly indicates that a particular condition or attribute is absent

Figure S2 shows the convergence of loss and accuracy during training of the three models This study uses the vgg-16 architecture, and the optimizers use the Adam optimization algorithm to fine-tune the parameters. Adam can iteratively update the neural network weights based on the training data, so it is suitable for solving optimization problems with large-scale data. The learning rate (learning rate) is 0.001, the X axis is the number of training steps (epoch), and the Y axis is the accuracy and loss value of model training. It can be seen from the figure that the accuracy of the final RGB and PCA is higher.

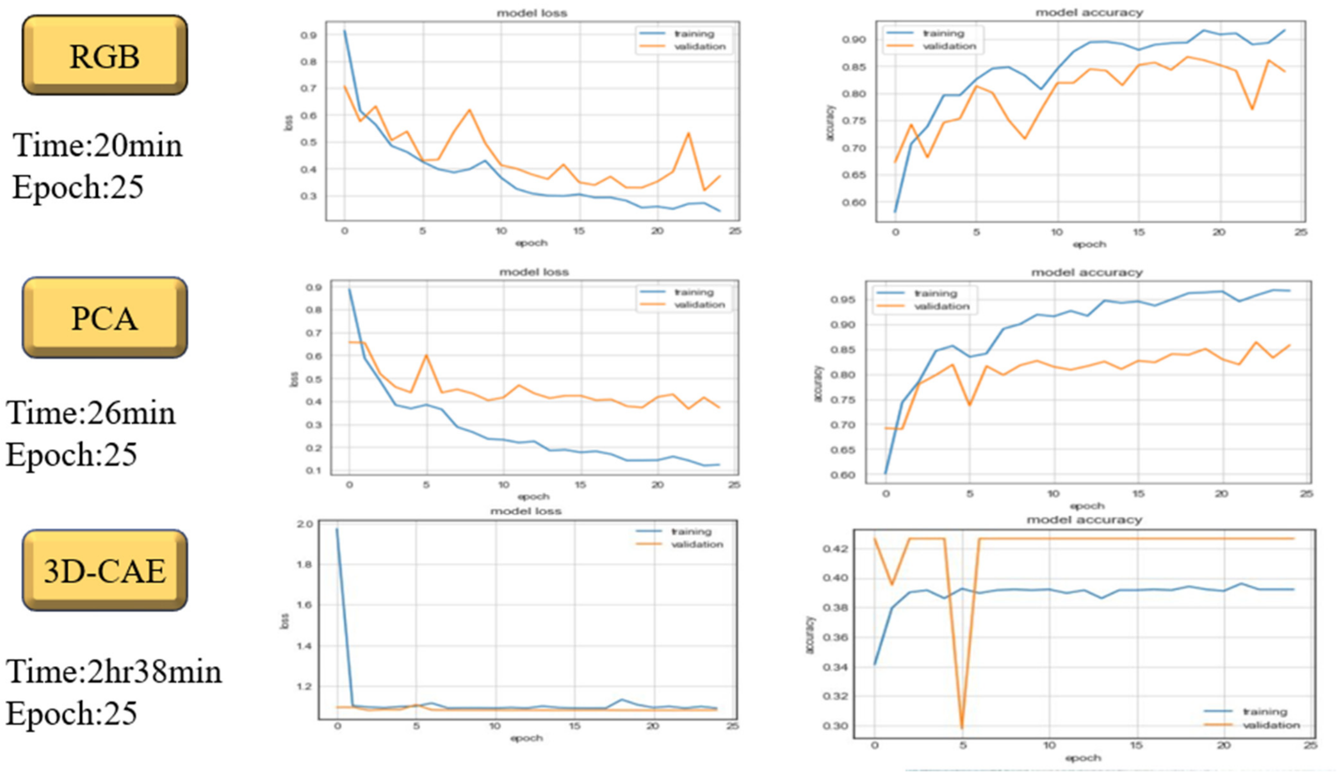

Figure S2. Convergence of loss and accuracy during training of the three models.

S2. Simulated Spectra and Color Difference

Figure S3 shows the difference between the measured and the simulated reflectance of the 6 important colors in the 24 color blocks. Additionally, it depicts the RMSE value of each of the color blocks. Figure S4 visualizes the actual difference between each of the color blocks. It shows the color differences between the measured and the simulated color blocks.

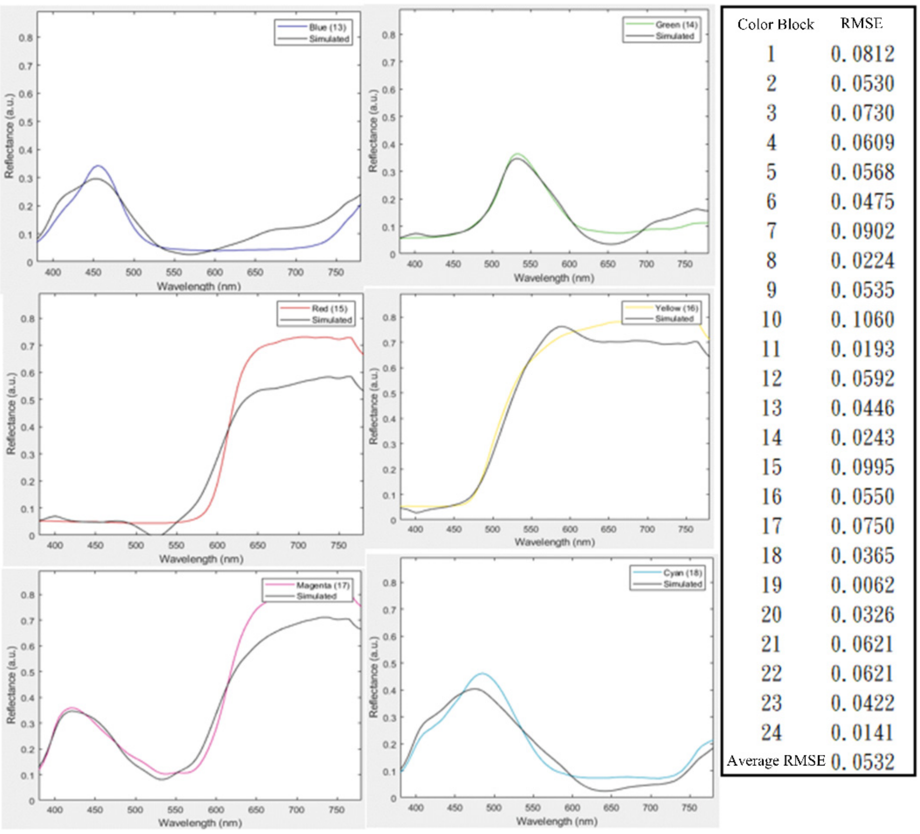

Color Block	RMSE
1	0. 0812
2	0. 0530
3	0. 0730
4	0. 0609
5	0. 0568
6	0. 0475
7	0. 0902
8	0. 0224
9	0. 0535
10	0. 1060
11	0. 0193
12	0. 0592
13	0. 0446
14	0. 0243
15	0. 0995
16	0. 0550
17	0. 0750
18	0. 0365
19	0. 0062
20	0. 0326
21	0. 0621
22	0. 0621
23	0. 0422
24	0. 0141
Average RMSE	0. 0532

Figure S3. The difference between the reflectance of the original 24 color blocks and the simulated color.

Measured Spectrum			Measured 24 Color Block	Simulated Spectrum			Simulated 24 Color Block	Color Difference
L*	a*	b*		L*	a*	b*		
37.608	13.65	24.56		35.072	12.54	26.36		2.6472
66.476	14.68	31.10		66.997	15.22	32.21		0.6377
50.440	-7.58	-6.44		50.662	-8.24	-8.39		1.4687
42.804	-16.14	30.50		45.201	-11.25	34.73		4.7667
56.157	5.70	-8.01		56.624	6.72	-5.68		2.3659
70.990	-34.14	16.44		71.136	-33.75	16.82		0.3223
61.973	32.36	66.76		61.934	32.14	63.80		0.9291
40.203	6.07	-27.03		39.573	5.11	-28.83		1.8064
51.588	46.04	27.52		50.879	46.20	27.14		0.7491
30.618	18.70	-9.45		32.559	18.18	-10.23		1.6666
72.242	-24.91	66.55		72.373	-24.67	66.33		0.1420
72.457	17.04	75.67		72.351	16.98	76.27		0.1887
29.176	13.90	-37.66		29.436	15.29	-35.11		2.3258
55.593	-40.93	42.88		55.105	-42.57	41.78		1.0031
41.659	53.78	34.95		42.553	53.36	36.59		1.2326
82.265	1.48	87.73		82.241	1.49	88.09		0.0756
51.287	46.36	1.08		51.308	46.29	0.76		0.1723
50.803	-31.41	-12.85		50.752	-31.25	-12.36		0.2906
95.467	-3.88	21.64		95.419	-3.75	21.77		0.1560
80.959	-3.08	18.47		81.083	-3.66	17.92		0.7511
66.384	-2.74	15.56		65.551	-3.00	14.31		1.0991
52.184	-2.26	12.86		51.605	-3.50	13.15		1.5847
36.473	-2.05	9.55		36.221	-2.15	7.16		1.7977
21.401	-1.45	6.28		20.484	-3.20	5.18		2.6205
							Average Difference	1.2833

Figure S4. The difference between the measured 24 color blocks and the simulated 24 color blocks using the developed HSI algorithm.

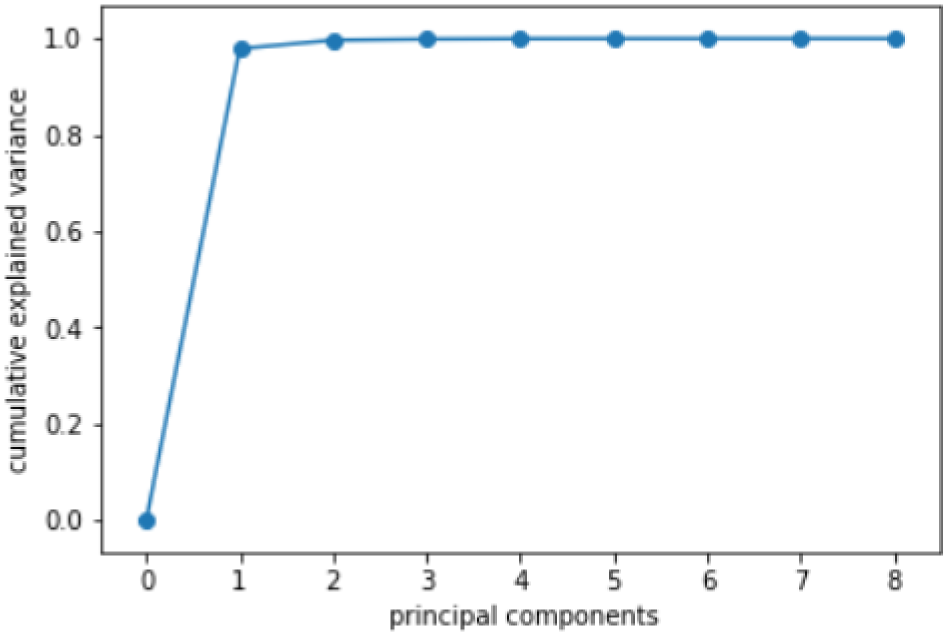

Figure S5. Cumulative explained variance vs. the number of Principal components.

As can be seen from the figure, it can be found in the spectral characteristics that only three principal components are used as the new base to represent 99.87% of the data, and most of the information of the original data is retained.
